# Uncovering the “Hidden” Relationship Between Old Age Assisted Dying and the Ancient Concept of Senicide

**DOI:** 10.3389/ijph.2025.1608760

**Published:** 2025-10-28

**Authors:** Uwe Güth, Shaun McMillan, Edouard Battegay

**Affiliations:** ^1^ Brust-Zentrum Zürich, Zurich, Switzerland; ^2^ Faculty of Medicine, University of Basel, Basel, Switzerland; ^3^ UZH Healthy Longevity Center, University Zurich, Zurich, Switzerland; ^4^ Merian Iselin Klinik, Basel, Switzerland

**Keywords:** assisted dying, senicide, burden to others, end-of-life-decision making process, geriatrics

## Abstract

**Objectives:**

To uncover a previously unrecognized link between the ancient cultural phenomenon of senicide, i.e. the practice of killing the elderly, and old age assisted dying (AD).

**Methods:**

Using an interpretative-phenomenological approach, the background of both phenomena is reviewed.

**Results:**

Senicide is based on utilitarian and altruistic judgements of the society and/or by older individuals themselves that they are no longer contributing members of the community. As a consequence, it is considered justifiable that the life of the elderly person should be intentionally ended. Similarly, in some cases of old age AD, those who express thoughts to die might feel a responsibility to relieve their family and society of the burden of their existence. When these aspects would take excess capacity in the decision-making process, AD might be considered a modern variant of senicide. Then, the question arises about how self-determined and autonomous the wish to die actually is and how much it is influenced by external pressures.

**Conclusion:**

The counseling team should openly address these motives to enable those with intentions to die and their families to engage in a more comprehensive decision-making process of AD.

## Introduction

Assisted dying (AD) is a comparatively new societal and medical phenomenon that has just started to become more prevalent in the Western world. In recent years, a growing number of countries, including Switzerland, Belgium, the Netherlands, Luxembourg, Austria, Spain, Canada, some US states (California, Colorado, Hawaii, Maine, Montana, New Jersey, New Mexico, Oregon, Washington state, Vermont, and the District of Columbia), New Zealand, and the six Australian states, conditionally allow for different forms of AD. Furthermore, legislation of AD is under active discussion in Germany, United Kingdom, France, Italy, Portugal, Ireland, and Slovenia. In so-called developing or emerging countries, AD is still not very widespread; only Colombia and, just last year, Ecuador have legalized AD. Today, based on the populations of the above mentioned countries and federal states, approximately 300 million persons worldwide have legal access to AD.

AD accounts for less than 1% of deaths in countries where it is limited to certain jurisdictions. In some countries, AD has been recently introduced, and thus long-term developments cannot yet be predicted. In countries with a longer history of legalized AD, increasing societal acceptance and more established infrastructures for implementing AD within national health systems, the proportion of deaths attributed to AD is reaching 5.4% in the Netherlands, 4.7% in Canada, 2.8% in Belgium and 2.4% in Switzerland (data from 2023 in each country) [1–4]. In these countries, a significant proportion involves the elderly and very elderly. In the Netherlands, the proportion of people aged ≥80 years at the time of their self-chosen death was 40% in 2024 [1]. Other countries also have similar AD age distributions (Canda: 39%; Belgium: 42%) [2, 3]. In Switzerland, the proportion of those aged over 80 is even higher at 55% [4]. This is also due to the influence of EXIT, the largest Swiss right-to-die organization, which provides support for approximately 70% of AD cases in the country. For many years, EXIT has been explicitly supporting AD for “persons suffering in and around old age” [5–7]. Due in part to these effective public relations, the category of *polymorbidity* ranks with 27% as the second most common reason for the desire to die among those receiving AD support from EXIT. Only those with cancer form an even larger indication group with 36% of cases (data from 2018 to 2022 [7]). Of late, the median age of those with indication of *polymorbidity* for AD was 89 years. This group obviously represents the phenomenon of “old age assisted dying” [6, 7].

In the above-mentioned cases, the “classic indication” of the *terminal illness requirement* for AD (which implies that the patient who seek for hastened death suffers from a severe disease that will lead to a natural death in the foreseeable future) is not met. In old age AD, a variety of age-related conditions influences the wish to terminate one owns life. These include functional incapacitations such as limited mobility, urinary incontinence, impaired vision and hearing loss, and social circumstances such as loneliness after the loss of a spouse, family or friends as well as institutionalization. In some cases, financial concerns, e.g., the cost of a long-term stay in a care facility, and the more general feeling of “just being a burden” on relatives may also play a role in the wish and the decision to die.

Throughout history, several ancient cultures, societies and ethnicities have accepted and approved of the killing of the elderly, i.e., *senicide* [8–10]. The background to senicide in each case was the assessment that older individuals are no longer a valuable and useful member to be carried through all the vicissitudes by the community.

Today, modern Western societies clearly do not overtly pressure their elderly to end their lives. However, in some cases of old age AD those who wish to die might also feel a responsibility to relieve their family and society of the burden of their existence [11–18]. If these utilitarian and altruistic components play too large a role in the decision to die, old age AD could be interpreted as a modern form of senicide. This deserves a closer look, because if utilitarianism and altruism carry too much weight in the elderly individuals’ decision-making towards AD, the question arises whether the intention to end their life is self-determined and autonomous and how much influence external family, social and economic pressure factors have on the request for AD. The Dutch philosopher and ethicist Govert den Hartogh may have implied such facets of decision-making by writing: “A personal choice is not necessarily a voluntary choice” [19].

It is a distinct mission of medical ethics to strive to uncover previously unrecognized connections between specific historical and cultural aspects in one perspective, and modern medicine in another. In conclusion, in this article we provide a link to clinical practice and discuss how an extended understanding of the “hidden” relationship between senicide and old age AD could lead to a more comprehensive counseling of elderly individuals expressing the wish to die.

## Method and Analysis

Using an interpretative-phenomenological approach, we illustrate how certain elements of the ancient concept of senicide might still be embedded in the modern practice of AD.

### Assisted dying and the shifting landscape of senicide: Dying as the last great act of self-determination and as a sacrificial death for the community?

Dr. Marion Schafroth, President of EXIT, does not currently see any social development towards an environment in which the elderly feel pressured to commit AD in Switzerland, where currently assisted suicide accounts for 2.4% of all deaths. She clearly places the autonomous decision of the individual at the forefront of the country’s AD culture (personal communication, statement authorized by the interviewee for this manuscript). However, the situation may look different in 10–15 years:

- Estimates suggest that around 5% of all deaths in Switzerland will then be caused by AD [20, 21]. While approximately 1,730 people currently choose AD (data from 2023), this may increase to around 3,500 in 15 years. With this, AD will become more mainstream and increasingly a social norm within the country’s culture of dying. The change in public awareness could in turn foster the number of cases [20, 21].
- A further social and political discussion will also weigh in: the financial burdens arising from pension payments and healthcare costs of an aging and over-aged population. It is highly likely that these economic aspects will be discussed more pointedly and publicly, with greater urgency, than today.

In a country where AD will be even more established as an option by the mid to late 2030s, it is then quite possible that many older people will not only see their AD as the last great act of autonomy, but also altruistically as a “benefit for the community” [8, 9].

This development could create a social climate in which the freedom to die is discreetly replaced by a moral obligation to use this freedom for the benefit of others and society at large. Such a utilitarian “euthanasia culture” could lead older people in need of care to feel pressured to assume their ultimate social responsibility and to relieve their relatives and society of the burden of their existence. In extreme cases, the ideal of a humane and independent end of life could mutate into *a duty to die*. [22–24] Former German President Johannes Rau contemplated about this development in his *Berlin Speech* already back in 2001: “Where continuing to live is only one of two legal options, anyone who imposes the burden of continuing to live on others becomes accountable. What appears to strengthen people’s self-determination can actually make them vulnerable to coercion.” [25].

The Japanese drama movie *Plan 75*, presented at the Cannes Film Festival in 2022 and selected as Japan’s entry for the Academy Awards’ *Best International Feature Film* category in 2023, converges in a similar direction [26–28]. In *Plan 75*, the Japanese government introduces a program that not only allows old people (those over 75) access to AD, but also facilitates it. A financial bonus paid out in advance can be used freely, and other end-of-life services, such as the organization of a free funeral or the termination of rental and other contracts, can be booked with employees of the program, who provide friendly advice to those willing to die. Nevertheless, senicide is not compulsory; every participant can leave the program at any time. With contemplative imagery and dialogue, the film depicts life situations of older people, characterized by feelings of isolation and resignation. The act of AD then appears to be an attractive option for combining both autonomous choice and social responsibility at the end of life. The film deliberately avoids futuristic effects and thus conveys a dystopian reality that does not seem to be so far ahead of our time.

It is no coincidence that Japanese created this movie. With a quarter of the population over 65 years of age, no other industrialized nation is ageing so rapidly [29–31]. Against the backdrop of a shrinking population with low birth rates and high life expectancy, there has been an on-going debate about how the state and society can cope with the financial burden of an ageing population with increasing pension payments and healthcare costs [29–31]. The country is far removed from the “obsession with staying young” of many Western countries, which often push older people to the margins of society. In contrast, Japanese truly and traditionally respect old age as an important and integral part their culture. This must make *Plan 75* all the more impressive in the country where it was written and directed.

### Dying as the last great act of self-determination …

*Old age assisted dying*, which might be considered a modern variant of senicide, will become significantly more important in the future due to social changes in Western countries. Countries having legalized AD with the indication of “unbearable suffering”, hence not restricted to the *terminal illness requirement*, will undergo an increase in the annual number of old age AD cases. An important and obvious factor in this development is that many people in ageing societies reach an advanced age, i.e., the number of potential eligible individuals for senicide/assisted dying is increasing. However, a far more important point is that the generation that will turn 80 or older in the next few years has been socialized in a completely different social environment than the generations of their parents and grandparents. While the latter were still far more trapped in norms shaped by origin, class, gender, religion and lack of access to education, many people who grew up later in the 1960s and 70s were able to shape their lives more freely with self-independence [32, 33]. Due to a shift in the cultural values, the increasing emphasis on individual autonomy to plan one’s life in modern society also extends to end-of-life decisions. Consequently, individuals who have exercised control over their lives may seek to shape their deaths from similar outsets, viewing AD as an appropriate option for them [34]. Many people of this generation, unlike those of previous generations, may not allow social conventions and norms to dictate how many deficiencies they may have to endure due to old age and illness [35]. The increasing volume of AD cases with the indication of age-related polymorbidity can therefore be seen as a symptom of a modern Western, increasingly secular society in which religious beliefs and tradition play a minor role in personal life decisions to a greater extent.

### … and as a sacrificial death for the community

Altruistic motivated suicide is a well-described subtype of suicide. Emil Durkheim, one of the founders of modern sociology, already defined this at the end of the 19th century in his groundbreaking and now considered classic study *Le suicide.* [36] Durkheim investigated the extent to which social factors influence the act of suicide. Durkheim saw suicide not only as an individual, psychologically explainable act, but also as a symptom of collective social deviance (in addition to altruistic suicide, he defined three other types: egoistic, anomic, and fatalistic).

Many people in Switzerland increasingly distance themselves from religious traditions and beliefs. However, Calvinism, an influential religious movement founded in the 16th century as part of the Swiss Reformation, remains an important factor in the country’s social and economic ethics [37–40]. This attitude most values the individual’s societal usefulness. When usefulness of a person declines, and the society is increasingly burdened by a person’s need of care, it is still considered a time-honored moral duty of society to care for a destitute individual. However, the social situation of being dependent on others is not welcome.

Altruistic expressions are frequently observed among the elderly. They no longer want to be burden anyone, even after death. Insurance companies have turned this into a business model: burial insurance policies are designed to address the rising costs of funerals and other end-of-life expenses, ensuring that these costs do not burden surviving family members. Another example for this exists in Sweden. The term *döstäda*, a word created from the Swedish words for *death/dying* and *tidying up*, has found its way into everyday language [41]. *Döstäda*, best translated into English as *death cleaning*, is defined as a self-determined and conscious act of decluttering and organizing the home before one’s own death in order to make it easier for loved ones or family members and not leave this work to the bereaved [42]. The concept of the “Gentle Art of Swedish Death Cleaning” has also been trending in popular culture, for example, in the book with the same title by Margareta Magnusson and in a recently broadcast American reality television series [43–45]. In both formats, however, the act of tidying up a house or apartment as a physical transformation takes a back seat. The focus is on *death cleaning* as a purifying, existential process that turns introspectively towards life, allowing individual self-reflection about what objects are truly necessary in life to keep and what you want others to remember you by [43, 45].

## Discussion

In a thoughtful public discourse on the subject of AD, we should also address utilitarian and altruistic considerations of euthanasia. Many advocates of AD tend to hold it in high regard as a free and autonomous decision at the end of life. In public deliberation, this “freedom rhetoric” has impressive effects. However, the critical debate would also need to deal with the potentially “dark”, economic-utilitarian side of reality. Of course, no one in Switzerland is currently pushing AD as an exit to the burden of an ageing society. However, the relative lack of societal criticism regarding AD, predominantly an old-age phenomenon, is noteworthy [46]. In a recent discussion, a colleague summed up the possible connection between the economic pressure of an ageing society and the fact that AD in old age is not unwelcome from an economic, and therefore social, point of view: “Who should get upset if a few old people kill themselves … in Switzerland?”

Numerous studies have investigated the motivation and motives of those seeking and willing to die with AD (overview in: [11]). The reasons to choose AD vary and depend on the underlying illness or situation. In the case of somatic illnesses, e.g., late-stage cancer, the physical symptoms associated with the illness, such as exhaustion, pain, nausea/vomiting and dyspnea, are often cited as relevant factors. However, social, psychological, existential and spiritual factors often play a role in the decision-making process. This applies particularly to situations in which there is no illness that would lead to natural death in the foreseeable future, namely, to old age AD. One important reason cited by many people that choose AD pertains to avoid being a burden on family and loved ones. The annual reports from Oregon, the US state with the longest tradition of euthanasia (the Oregon Death with Dignity Act came into force in 1997), show *Burden on family, friends/caregivers* as the fourth most frequently cited existential reason for seeking AD at 47% [47]. The other end-of-life concerns cited more often by an individuals’ intent to die were *loss of autonomy, decreasing ability to participate in activities that made life enjoyable* (90%, respectively) and *loss of dignity* (70%).

Some of those studies that have examined the motivations of people who request AD and also evaluated psychological and existential factors, listed *to spare others from the burden of oneself* [11] as one of several influencing factors in the decision-making process [11, 13, 16–18]. In the majority of the publications on AD, however, altruistic concerns were not considered as an influencing factor. From this, one can also conclude that these aspects, in line with a blind spot, might have also been neglected by those who counseled the people who wished to die and thus were not actively addressed in the decision-making process.

Counseling elderly people who are making a choice to die is a demanding task. The complexity of the task is often increased by the fact that family members are also involved in the decision-making process for or against AD [48–50]. This requires a high level of medical knowledge from those who provide it. In addition, and most importantly, it requires a high degree of sensitivity and maturity acquired through life experience and in sensing open and hidden agendas of patients.

Maintaining autonomy, personal dignity and self-determination are important existential determinants in the decision-making process for AD. However, it is important to understand that neglecting utilitarian and altruistic motives might lead to inadequate counseling of elderly individuals who wish to end their life. With a comprehensive shared decision-making process of AD, these potentially hidden motives should openly be addressed empowering the persons expressing thoughts to die and their families to develop an awareness with a clarity whether and to what extent these motives are included in the decision to die.
